# Influence of dietary patterns and physical activity on bone mineral content and density, osteoporosis among children with stimulant use

**DOI:** 10.3389/fped.2022.976258

**Published:** 2022-09-21

**Authors:** Qianqian Li, Jiaxiu Zhou

**Affiliations:** ^1^Department of Psychological Counseling, The Second Affiliated Hospital of Chongqing Medical University, Chongqing, China; ^2^Child Health and Mental Health Center, Shenzhen Children’s Hospital, Shenzhen, China

**Keywords:** dietary patterns, physical activity, BMD, BMC, osteoporosis, stimulant use

## Abstract

**Aim:**

To examine the relationship between dietary patterns (DPs) and physical activity (PA) on bone mineral content (BMC), bone mineral density (BMD), and osteoporosis in children with stimulant use.

**Methods:**

A cross-sectional study collected information on participants from the National Health and Nutrition Examination Survey (NHANES) *via* multistage stratified sampling. The baseline variables included the following: age, gender, the dietary approaches to stop hypertension (DASH) score, the Mediterranean diet (MD) score, and the Alternative Healthy Eating Index-2010 (AHEI-2010). The univariate and multivariate linear-regression analyses were carried out to explore the statistical correlation between the DPs and PA on BMC and BMD in children with stimulant use or non-stimulant use. In addition, we also investigated the association between DPs and PA on osteoporosis *via* logistic regression analyses.

**Results:**

A total of 6,294 participants were eligibly enrolled in this study eventually. After adjusting age, gender, body mass index (BMI), race, family income, serum 25-hydroxyvitamin D, and serum cotinine, the multivariate linear-regression analysis showed that the MD was positively associated with total femur BMD, total femur BMC, femoral neck BMD, and femoral neck BMC among stimulant use group; high PA was associated with total femur BMD, total femur BMC, femoral neck BMD, femoral neck BMC, lumbar spine BMD, lumbar spine BMC and osteoporosis in stimulant use group.

**Conclusion:**

Improved adherence to MD, DASH, AHEI-2010 or increased physical activity may increase BMD, BMC and reduce the risk of osteoporosis; children with stimulant use should improve their adherence to the MD and do more PA compared with children without stimulant use.

## Introduction

Osteoporosis is a kind of common systemic skeletal disease and its prevalence increases with age, it could result in enhanced bone fragility and risk of fracture, which became an emerging public health issue worldwide (1). Existing research have recognized that low bone mineral density (BMD) is associated with a higher risk for fragility fracture and a hallmark of osteoporosis (2, 3). Peak bone mass is an important determinant of BMD throughout life. A growing body of scientific evidence showed that optimizing bone mass accumulation during childhood may be of great importance in decreasing the risk of osteoporosis in later life (4, 5).

An epidemiological study reported that 60–80% of the variability in BMD was attributed to genetic determinants (6), but, several modifiable factors, such as dietary and physical activity (PA), play important roles in modulating bone gain during childhood and adolescence (7, 8). Most approaches to bone gain are focused on nutrients, such as calcium or vitamin D. In a similar manner, fruits and vegetables showed beneficial effects (9, 10). It is worth noting that the effect of a single nutrient may be too small to detect; therefore, dietary pattern (DP) is a more comprehensive approach to explaining the relationship between overall diet and bone gain than individual foods or nutrients (11). Previous studies had been shown that the contribution of PA to bone development during growth (12–14). The PA increased bone formation by promoting mechanical forces applied to bones, and high-intensity PA improved bone mineral accumulation in children and adolescents (13).

Attention deficit hyperactivity disorder (ADHD) is a common neurodevelopmental disorder worldwide (15), which has affected the physical and mental health of children to adults. At present, the stimulants have been recognized as the most commonly used medication for ADHD (16). Several studies have proposed that stimulants can not only slow down children’s growth rates but also affect their bone health (16, 17). A cross-sectional analysis by Feuer et al. showed that the use of stimulants might decrease bone mass compared to children who did not use stimulants, and the possible mechanism could be explained by the fact that stimulants stimulated sympathetic activation *via* norepinephrine, which decreases the bone mass (16). Therefore, it is critical to pay attention to bone mass in children using stimulant drugs. However, to our knowledge, there are few studies about the influence of adherence to dietary patterns (DPs) and PA on bone outcomes in children with stimulant use so far. Herein, the aim of this cross-sectional study is to examine the relationship between DPs and PA on bone mineral content (BMC), BMD, and osteoporosis in children with stimulant use.

## Materials and methods

### Study setting and participants

The National Health and Nutrition Examination Survey (NHANES) is a stratified, multistage program of studies conducted periodically by the National Center for Health Statistics (NCHS) and the Centers for Disease Control (CDC) and Prevention, aimed at assessing the health and nutritional status of adults and children in the United States (18, 19). The examination contents included physiological measurements, questionnaires on health and nutrition, and laboratory tests.^1^

This cross-sectional study collected information from 22,475 participants between 2005 and 2010, 2013 and 2014, and 2017 and 2018 from the NHANES database *via* multistage stratified sampling. Participants aged 8–20 years who underwent bone mineral density measurements were included in this study. At the same time, we excluded some participants without bone mineral density measurements (*n* = 15,986) and missing dietary scores (*n* = 195). All the participants provided written consent to participate in the NHANES survey, and data collection was approved by the National Center for Health Statistics (NCHS) Research Ethics Review Committee.

### Data collection

#### Definition of osteoporosis

The International Society of Clinical Densitometry (ISCD) guidelines, pointed out that the diagnosis of osteoporosis in children is defined by the presence of both, a clinically significant fracture history [one or more long bone fractures or vertebral compression fracture (VF)] and a low BMC or BMD (low BMC or BMD is defined as a BMC or areal BMD Z-score ≤ –2.0) (20, 21). The dual-energy X-ray absorptiometry (DXA) was used to measure total femur BMD (mg/cm^2^), femoral neck BMD (mg/cm^2^), lumbar spine BMD (mg/cm^2^), total femur BMC (mg), femoral neck BMC (mg), and lumbar spine BMC (mg), and osteoporosis.

#### Definition of dietary patterns

In this study, DPs contained the dietary approaches to stop hypertension (DASH) score, the Mediterranean diet (MD) score, and the Alternative Healthy Eating Index-2010 (AHEI-2010).

Dietary approaches to stop hypertension adherence score was calculated following the method by Fung et al. (22), which was based on foods and nutrients namely, fruits, vegetables, nuts and legumes, whole grains, low-fat dairy, sodium, red and processed meats, and sweetened beverages. The participants received 1 point for those in the lowest quintile and 5 points for those in the highest quintile of intakes of fruit, vegetables, nuts and legumes, low-fat dairy products, and whole grains, according to the quintile rankings. Those with the lowest quintile of intake of sodium, sugar-sweetened beverages, and red and processed meats received a score of 5 and those in the highest quintile received a score of 1. The scores were totaled with a possible score range from 8 (lowest adherence) to 40 (greatest adherence).

According to the method of Sofi et al. (23), adherence to MD scores was calculated, for fruits, vegetables, whole grains, legumes, nuts, legumes, and fish, “0”, “1”, “2” points for the lowest, middle, highest category, separately. In contrast, for red meat and meat products, “2”, “1”, “0” points for the lowest, middle, highest category, separately; and for alcohol, which gets a score of “0” for >24 g, “1” for <12 g, and “2” for 12–24 g of intake per day. The MD scores in this study were calculated by using gram intakes. However, the fruits and vegetable variables were reported as cup equivalent (CE) intakes, as gram intakes were not available, the score for vegetables and fruits was calculated by referring to K. Taylor’s method in our study (24), namely, fruit scores of MD were: “0” for < 1 CE, “1” for ≥ 1 CE, and “2” for ≥ 2 CEs per day, MD vegetables received the score of “0” = < 0.5 CEs, “1” = ≥ 0.5 CEs, and “2” = ≥ 1 CE per day, respectively. The score for grams of olive oil intake was “0” for 14 g, “1” for ≥ 14 g, and “2” for ≥28 g per day.

AHEI-2010 (25): The participants received a score between 0 (minimal adherence) and 10 (maximal adherence) for each component, based on dietary intake. A sum of all components was calculated and ranged from 0 (poorest dietary quality) to 110 (highest dietary quality), with a higher score representing better adherence.

#### Definition of physical activity

On the basis of the PA Guidelines for Americans, PA was divided into four categories (26): sedentary (doing no regular PA), insufficient (doing some regular PA, but not meeting the minimum standards of the guidelines), moderate (500–1,000 MET-minutes of PA per week), and high (≥1,000 MET-minutes of PA per week).

#### Stimulant use

The data on stimulant use was obtained from self-reports (16). Children with stimulant use were defined as participants who reported use of amphetamine, dextroamphetamine, methamphetamine, methylphenidate, dexmethylphenidate, or lisdexamfetamine. Children without stimulant use were defined as participants who did not report the use of these medications.

#### Covariates

The baseline variables included the following: age, gender, body mass index (BMI, kg/m^2^), race, family income, serum 25-hydroxyvitamin D (nmol/L), and serum cotinine (ng/mL). The BMI is calculated by dividing weight (kg) by height (m^2^) squared. The sensitivity analysis of the missing data before and after interpolation was showed in Table 1.

**TABLE 1:** The sensitivity analysis of missing data before and after interpolation.

Variates	Ratio of missing values (%)	Before the interpolation	After the interpolation	Statistics	*P*
BMI, kg/m^2^, Mean (SE)	0.17	22.54 ± 5.72	22.54 ± 5.72	*t* = –0.001	0.997
Race, *n* (%)	0.00				
Family income, *n* (%)	0.76			χ^2^ = 0.111	0.739
<20000$		1520 (24.15)	1504 (23.90)		
≥20000$		4774 (75.85)	4790 (76.10)		
Serum cotinine, M (Q_1_, Q_3_)	10.04	0.06 (0.04, 0.50)	0.06 (0.04, 0.53)	Z = 0.431	0.666

BMI, body mass index.

### Statistical analysis

Under the weighted large sample, the measurement data were approximately normal distribution, and were described by mean (standard error) (SE), and adopted the independent sample *t*-test to perform a comparison between groups. The enumeration data were described as the number of cases and composition ratio *n* (%), the comparison between groups *via* Chi-square or Fisher’s exact test.

The univariate and multivariate linear-regression analyses were carried out to explore the statistical correlation between the DPs and PA on BMC and BMD in children with stimulant use or non-stimulant use. In addition, we investigated the association between DPs and PA on osteoporosis in children with stimulant use and non-stimulant use, respectively, *via* logistic regression analyses. Two models were adopted in our study, Model 1 was the coarse model, representing the unadjusted variable. Model 2 adjusted age, gender, BMI, race, family income, serum 25-hydroxyvitamin D, and serum cotinine. Statistical analyses were conducted by SAS (version 9.4) software; the missing values were interpolated by R software (version 4.20). All statistical tests were performed by using bilateral tests. *P* < 0.05 was regarded as statistically significant.

## Results

### Baseline characteristics

Among the total 22,475 participants, 6,294 participants were eligible to enroll in this study eventually. These subjects were divided into the non-stimulant use group (*n* = 6,072) and the stimulant use group (*n* = 222) based on whether they used stimulants. In this population, the average age was 13.93 y, of which the subjects consisted of 3,257 boys (52.11%) and 3,037 girls (47.89%), the mean scores of MD, DASH, and AHEI-2010 were 6.17, 23.82, and 25.54, respectively, more than half of the people had a high level of PA. In addition, we also found that the total femur BMD, femoral neck BMD, lumbar spine BMD, total femur BMC, femoral neck BMC, lumbar spine BMC, and the mean scores of MD and AHEI-2010 of subjects with stimulant use were significantly lower compared to the non-stimulant use group, not only that, for the non-stimulant use group, 51.33% had the high level of PA, however, only 48.75% had the high level of PA among stimulant use group. Detailed baseline information was given in Table 2.

**TABLE 2:** Differences between stimulant use group and non-stimulant use group.

Variates	Total (*n* = 6294)	Non-stimulant use group (*n* = 6,072)	Stimulant use group (*n* = 222)	Statistics	*P*
Age, years, Mean (SE)	13.93 (0.07)	13.98 (0.07)	12.87 (0.33)	*t* = 3.42	0.001
**Gender, n (%)**				χ^2^ = 13.550	<0.001
Male	3,257 (52.11)	3,102 (51.27)	155 (69.72)		
Female	3,037 (47.89)	2,970 (48.73)	67 (30.28)		
BMI, kg/m^2^, Mean (SE)	22.19 (0.11)	22.26 (0.11)	20.79 (0.42)	*t* = 3.28	0.002
**Race, *n* (%)**				χ^2^ = 24.027	<0.001
Mexican American	1,821 (13.02)	1,787 (13.36)	34 (5.95)		
The other races	892 (12.76)	858 (12.92)	34 (9.35)		
Non-Hispanic whites	1,885 (60.06)	1,777 (59.35)	108 (74.97)		
Non-Hispanic blacks	1,696 (14.16)	1,650 (14.37)	46 (9.73)		
Family income, *n* (%)				χ^2^ = 0.455	0.500
<20,000$	1,520 (17.97)	1,464 (17.87)	56 (20.12)		
≥20,000$	4,774 (82.03)	4,608 (82.13)	166 (79.88)		
Serum 25-hydroxyvitamin D, nmol/L, Mean (SE)	66.11 (0.83)	65.66 (0.82)	75.42 (1.87)	*t* = –5.79	<0.001
Serum cotinine, ng/mL, Mean (SE)	13.94 (1.09)	14.47 (1.13)	2.93 (1.34)	*t* = 6.33	<0.001
**Total femur, Mean (SE)**					
BMD	0.91 (0.00)	0.91 (0.00)	0.83 (0.01)	*t* = 5.69	<0.001
BMC	29.17 (0.24)	29.33 (0.25)	25.73 (1.00)	*t* = 3.40	0.001
Femoral neck, Mean (S.E)					
BMD	0.84 (0.00)	0.84 (0.00)	0.77 (0.01)	*t* = 5.25	<0.001
BMC	4.08 (0.03)	4.10 (0.03)	3.69 (0.10)	*t* = 3.95	<0.001
**Lumbar spine, Mean (SE)**					
BMD	0.91 (0.00)	0.91 (0.00)	0.81 (0.02)	*t* = 3.96	<0.001
BMC	46.18 (0.42)	46.47 (0.44)	40.20 (1.52)	*t* = 5.67	<0.001
MD, Mean (SE)	6.17 (0.06)	6.17 (0.06)	6.20 (0.17)	*t* = –0.17	0.868
DASH, Mean (SE)	23.82 (0.11)	23.82 (0.12)	23.80 (0.29)	*t* = 0.08	0.937
AHEI-2010, Mean (SE)	25.54 (0.19)	25.62 (0.19)	23.91 (0.54)	*t* = 3.13	0.003
**Physical activity, *n* (%)**				χ^2^ = 6.595	0.086
Sedentary	1,941 (28.72)	1,865 (28.51)	76 (33.23)		
Insufficient	603 (9.56)	591 (9.78)	12 (4.88)		
Moderate	639 (10.51)	610 (10.38)	29 (13.13)		
High	3,111 (51.21)	3,006 (51.33)	105 (48.75)		

BMI, body mass index; BMD, bone mineral density; BMC, bone mineral content; MD, Mediterranean diet; DASH, dietary approaches to stop hypertension; AHEI-2010, Alternative Healthy Eating Index-2010.

### The association of dietary patterns and physical activity on bone mineral content, bone mineral density in children with stimulant use and non-stimulant use

The univariate linear-regression analysis was used to explore the possible confounding factors in the association of the DPs and PA on BMC and BMD in children. Table 3 indicated that age, gender, BMI, race, family income, serum 25-hydroxyvitamin D, and serum cotinine may be the confounding factors (*P* < 0.05). After adjusting for confounding factors, we adopted the multivariate linear-regression analysis to assess the relationship of DPs and PA on BMC and BMD in children with stimulant use and non-stimulant use. Table 4 showed the differences in the association of DPs and PA on BMD and BMC between the non-stimulant use group and stimulant use group. For stimulant use group, the MD was positively associated with total femur BMD, total femur BMC, femoral neck BMD, and femoral neck BMC [β = 8.75, 95% confidence interval (CI):4.90–32.59; β = 1.44, 95% CI: 0.41–2.46; β = 5.38, 95% CI: 2.19–28.56; β = 0.14, 95% CI: 0.04–0.25], but there was no statistical correlation among non-stimulant use group (*P* > 0.05), except the total femur BMC (β = 0.29, 95% CI:0.03–0.55; *P* < 0.05). In addition, there was a positive correlation between the moderate and high levels of PA and total femur BMD, total femur BMC, femoral neck BMD, femoral neck BMC, lumbar spine BMD, and lumbar spine BMC among the non-stimulant use group. However, only high PA was associated with total femur BMD, total femur BMC, femoral neck BMD, femoral neck BMC, lumbar spine BMD, and lumbar spine BMC in the stimulant use group (*P* < 0.05).

**TABLE 3:** Univariate linear-regression analysis of covariates.

Variates	Total femur				Femoral neck				Lumbar spine			
	BMD		BMC		BMD		BMC		BMD		BMC	
	β (95% CI)	*P*	β (95% CI)	*P*	β (95% CI)	*P*	β (95% CI)	*P*	β (95% CI)	*P*	β (95% CI)	*P*
Age	34.93 (33.45, 36.41)	<0.001	2.25 (2.16, 2.35)	<0.001	29.60 (28.15, 31.05)	<0.001	0.23 (0.22, 0.24)	<0.001	45.25 (43.87, 46.63)	<0.001	3.84 (3.67, 4.00)	<0.001
Gender (female)	–65.84 (–78.59, 53.09)	<0.001	–7.26 (–7.95, 6.58)	<0.001	–53.23 (–63.40, 43.06)	<0.001	–0.69 (–0.77, 0.61)	<0.001	49.99 (36.43, 63.55)	<0.001	–4.41 (–5.59, 3.23)	<0.001
BMI	19.46 (18.49, 20.44)	<0.001	1.05 (0.99, 1.12)	<0.001	18.54 (17.60, 19.49)	<0.001	0.13 (0.12, 0.13)	<0.001	21.40 (20.26, 22.55)	<0.001	1.55 (1.43, 1.66)	<0.001
**Race, *n* (%)**												
Mexican American	Ref		Ref		Ref		Ref		Ref		Ref	
The other races	–88.54 (–103.74, 73.34)	<0.001	–3.65 (–4.47, 2.83)	<0.001	–82.06 (–96.36, 67.76)	<0.001	–0.46 (–0.55, 0.37)	<0.001	–89.32 (–107.06, 71.59)	<0.001	–6.12 (–7.63, 4.61)	<0.001
Non-Hispanic whites	–106.99 (–128.21, 85.77)	<0.001	–4.45 (–5.59, 3.31)	<0.001	–101.18 (–121.46, 80.90)	<0.001	–0.53 (–0.64, 0.41)	<0.001	–85.15 (–110.20, 60.09)	<0.001	–5.46 (–7.37, 3.55)	<0.001
Non-Hispanic blacks	–73.82 (–90.19, 57.45)	<0.001	–1.60 (–2.52, 0.68)	0.001	–72.97 (–88.45, 57.48)	<0.001	–0.23 (–0.33, 0.13)	<0.001	–58.53 (–77.47, 39.59)	<0.001	–1.97 (–3.64, 0.29)	0.022
Family income (ł20,000$)	–17.64 (–33.23, 2.05)	0.027	–0.64 (–1.61, 0.33)	0.192	–18.03 (–32.04, 4.02)	0.013	–0.09 (–0.19, 0.01)	0.081	–27.04 (–46.70, 7.39)	0.008	–1.70 (–3.31, 0.08)	0.040
Serum 25-hydroxyvitamin D	–0.85 (–1.14, 0.56)	<0.001	–0.03 (–0.05, 0.01)	0.001	–0.84 (–1.10, 0.57)	<0.001	–0.01 (–0.01, 0.01)	<0.001	–1.13 (–1.44, 0.81)	<0.001	–0.06 (–0.09, 0.03)	<0.001
Serum cotinine	0.62 (0.48, 0.75)	<0.001	0.05 (0.04, 0.05)	<0.001	0.53 (0.39, 0.67)	<0.001	0.01 (0.01, 0.01)	<0.001	0.79 (0.69, 0.89)	<0.001	0.08 (0.07, 0.09)	<0.001

BMI, body mass index; BMD, bone mineral density; BMC, bone mineral content.

**TABLE 4:** The association of dietary patterns and physical activity on BMC, BMD in children with stimulant use and non-stimulant use.

Variates	Total femur				Femoral neck				Lumbar spine			
	BMD		BMC		BMD		BMC		BMD		BMC	
	β (95% CI)	*P*	β (95% CI)	*P*	β (95% CI)	*P*	β (95% CI)	*P*	β (95% CI)	*P*	β (95% CI)	*P*
**Non-stimulant use**												
MD	3.35 (–1.85, 8.55)	0.202	0.29 (0.03, 0.55)	0.031	2.24 (–3.12, 7.60)	0.405	0.02 (–0.02, 0.06)	0.284	0.09 (–4.72, 4.90)	0.971	0.35 (–0.23, 0.92)	0.230
DAS H	1.34 (–3.24, 5.93)	0.559	0.15 (–0.08, 0.39)	0.185	1.46 (–2.96, 5.88)	0.509	0.02 (–0.01, 0.05)	0.232	5.26 (1.04, 9.48)	0.016	0.08 (–0.44, 0.60)	0.758
AHEI-2010	4.90 (0.38, 9.42)	0.034	0.51 (0.26, 0.75)	<0.001	4.59 (0.14, 9.05)	0.043	0.05 (0.03, 0.08)	<0.001	6.55 (2.33, 10.77)	0.003	0.70 (0.25, 1.16)	0.003
** Physical activity**												
Sedentary	Ref		Ref		Ref		Ref		Ref		Ref	
Insufficient	10.5 (–5.37, 26.37)	0.190	3.61 (3.08, 4.13)	<0.001	5.8 (–9.44, 21.04)	0.448	0.07 (–0.03, 0.17)	0.175	16.59 (–0.57, 33.75)	0.058	4.76 (3.78, 5.74)	<0.001
Moderate	31.87 (18.3, 45.44)	<0.001	2.13 (1.33, 2.93)	<0.001	26.29 (12.67, 39.92)	<0.001	0.2 (0.11, 0.3)	<0.001	31.05 (17.79, 44.31)	<0.001	3.18 (1.37, 4.99)	0.001
High	58.4 (46.98, 69.82)	<0.001	3.61 (3.08, 4.13)	<0.001	46.93 (36.4, 57.46)	<0.001	0.36 (0.3, 0.43)	<0.001	47.45 (37.4, 57.49)	<0.001	4.76 (3.78, 5.74)	<0.001
**Stimulant use**												
MD	8.75 (4.90, 32.59)	0.009	1.44 (0.41, 2.46)	0.007	5.38 (2.19, 28.56)	0.023	0.14 (0.04, 0.25)	0.010	13.85 (–3.80, 31.49)	0.121	1.00 (–0.38, 2.39)	0.152
DASH	–5.32 (–25.47, 14.84)	0.598	0.03 (–1.17, 1.24)	0.958	–1.87 (–20.01, 16.28)	0.837	0.01 (–0.12, 0.13)	0.928	–4.34 (–28.56, 19.87)	0.719	0.04 (–1.70, 1.78)	0.961
AHEI-2010	12.94 (–6.32, 32.20)	0.183	1.47 (–0.01, 2.94)	0.051	7.40 (–10.48, 25.28)	0.409	0.10 (–0.04, 0.25)	0.161	8.55 (–15.17, 32.28)	0.471	1.28 (–0.64, 3.20)	0.186
** Physical activity**												
Sedentary	Ref		Ref		Ref		Ref		Ref		Ref	
Insufficient	22.86 (–82.31, 129.8)	0.669	1.26 (–3.2, 5.71)	0.572	16.65 (–93.99, 127.3)	0.763	0.05 (–0.53, 0.64)	0.859	17.33 (–68.73, 103.3)	0.687	1.00 (–6.12, 8.13)	0.778
Moderate	–12.89 (–82.31, 56.53)	0.710	–0.75 (–3.63, 2.12)	0.600	–11 (–72.03, 50.02)	0.718	–0.09 (–0.44, 0.25)	0.583	–10.2 (–73.04, 52.64)	0.745	–0.37 (–4.84, 4.1)	0.869
High	80.2 (32.5, 127.9)	0.002	5.16 (2.09, 8.24)	0.002	83.44 (36.08, 130.81)	0.001	0.5 (0.19, 0.82)	0.003	74.55 (16, 133.11)	0.014	6.67 (2.72, 10.62)	0.001

BMD, bone mineral density; BMC, bone mineral content; MD, Mediterranean diet; DASH, dietary approaches to stop hypertension; AHEI-2010, Alternative Healthy Eating Index-2010; β, the standardized regression coefficient. Adjusted age, gender, BMI, race, family income, serum 25-hydroxyvitamin D, and serum cotinine.

### The association of dietary patterns and physical activity on osteoporosis among children with stimulant use and non-stimulant use

**FIGURE 1:**
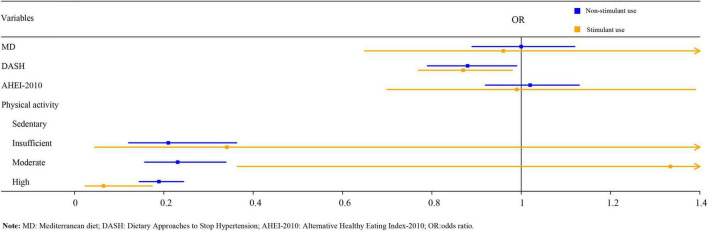
The forest graph of dietary patterns, physical activity, and osteoporosis in children.

As illustrated in Table 5, logistic regression analysis suggested that after adjusting for age, gender, BMI, race, family income, serum 25-hydroxyvitamin D, and serum cotinine, DASH score, insufficient, moderate, and high PA might be related to a reduced risk of osteoporosis in the non-stimulant use group [odds ratio (OR) = 0.87, 95%CI:0.77–0.98, *P* < 0.05; OR = 0.21, 95%CI:0.12–0.36, *P* < 0.001; OR = 0.23, 95%CI:0.16–0.34, *P* < 0.001; OR = 0.19, 95%CI:0.15–0.24, *P* < 0.001). It is worth noting that, only high PA was statistically associated with a reduced risk of osteoporosis in the stimulant use group (OR = 0.07, 95%CI: 0.02–0.17, *P* < 0.001). The forest plot of DPs and PA on osteoporosis among children with stimulant use and non-stimulant use was exhibited in Figure 1.

**TABLE 5:** The association of dietary patterns and physical activity on osteoporosis among total children, children with stimulant use and non-stimulant use.

Variates	Model 1		Model 2	

	**OR (95% CI)**	***P***	**OR (95% CI)**	***P***
**Non-stimulant use**				
MD	1.03 (0.93–1.15)	0.534	1.00 (0.89–1.12)	0.980
DASH	0.78 (0.69–0.88)	<0.001	0.87 (0.77–0.98)	0.021
AHEI-2010	0.94 (0.86–1.03)	0.185	1.02 (0.92–1.13)	0.684
** Physical activity**				
Sedentary	Ref		Ref	
Insufficient	0.19 (0.12–0.32)	<0.001	0.21 (0.12–0.36)	<0.001
Moderate	0.24 (0.16–0.36)	<0.001	0.23 (0.16–0.34)	<0.001
High	0.19 (0.15–0.24)	<0.001	0.19 (0.15–0.24)	<0.001
**Stimulant use**				
MD	0.90 (0.61–1.33)	0.591	0.96 (0.65–1.41)	0.816
DASH	1.01 (0.74–1.36)	0.960	0.97 (0.70–1.33)	0.837
AHEI-2010	0.99 (0.71–1.37)	0.942	0.99 (0.70–1.39)	0.938
** Physical activity**				
Sedentary	Ref		Ref	
Insufficient	0.39 (0.04–4.02)	0.412	0.34 (0.05–2.60)	0.282
Moderate	1.34 (0.40–4.57)	0.623	1.33 (0.37–4.88)	0.652
High	0.07 (0.03–0.18)	<0.001	0.07 (0.02–0.17)	<0.001

MD, Mediterranean diet; DASH, dietary approaches to stop hypertension; AHEI-2010, Alternative Healthy Eating Index-2010.

Model 1: unadjusted.

Model 2: adjusted age, gender, BMI, race, family income, serum 25-hydroxyvitamin D, and serum cotinine.

### Discussion

In this study, we investigated the influence of DPs and PA on BMC, BMD and osteoporosis in children with stimulant use and non-stimulant use. The study found that adherence to the MD and high PA had positively associated with BMD and BMC, and only high PA may be correlated with a decreased risk of osteoporosis among children with stimulant use. Our finding indicated that children with stimulant use should be more concerned about the role of high PA on bone health.

In general, stimulants are the most commonly used psychopharmacologic drugs to treat ADHD. However, stimulants could increase sympathetic tone and affect bone remodeling (16). Attention to the bone health of children with stimulant use is critical. Previous studies have reported that PA is one of the most important factors affecting peak bone mass during childhood, which is consistent with our findings. High PA could increase the BMC and BMD, and decrease the risk of osteoporosis among children with or without stimulant use. One review reported the influence of genetic, fetal, and environmental on bone mass acquisition among healthy children, and indicated the importance of PA in the process of bone mass acquisition (27). Mechanical signals, as the main component of PA, could promote anabolism in bones and muscles. Dynamic ground-reaction force generated by PA transmit a series of signals in bone and muscle tissue, making the cells, tissues, and organs to mechanically strain and accelerate, thereby causing increased muscle mass, bone mineralization, and reduced systemic inflammation (28). In a similar manner, a high level of PA has also been shown to be closely related to effectively improving BMD, even in people with a poor genetic predisposition in development of bone. One study has confirmed that high PA in early life might enhance hip strength and prevent osteoporosis later in life (29). Not only that, Marin-Puyalto et al. also reported that vigorous PA played a pivotal role in improving BMC and BMD among adolescent boys, with a greater benefit if they keep the periods of vigorous PA longer or more frequent (30). These studies showed that high PA seems to be one of the most effective means of bone growth, which suggested that children should be encouraged to do high physical activity as appropriate, such as participating in group activities and outdoor activities, learning sports, and developing the habit of exercising.

It is well known that MD is characterized by rich in fruits and vegetables, cereals, small amounts of red meat, lower sugar, and saturated fatty acids, which has attracted great attention in terms of its impact on human health. A meta-analysis showed that the adherence to MD was associated with a reduced risk of fracture and higher mean BMD, this may be due to the greater adherence to MD might decrease the concentration of pro-inflammatory cytokines (such as C-reactive protein and interleukin-6), which caused the lower rate of bone resorption (31). Although several studies suggested that adherence to MD was related to a lower incidence of osteoporosis and increased BMD, few studies assessed the association of MD and BMD, BMC to date among children with stimulant use. In the study, the adherence to MD was associated with total femur BMD, total femur BMC, femoral neck BMD, and femoral neck BMC in children with stimulant use, but it’s worth noting that MD was not statistically correlated with lumbar spine BMD and BMC, this is inconsistent with previous research (31), which is probably because of the different study populations included, lumbar spine BMD and BMC in children were significantly affected by their height and BMI (32). In addition, our study found that DASH adherence was associated with lumbar spine BMD in children without stimulant use but not in children with stimulant use. In a cross-sectional study by Noel et al. (33), they also reported that DASH was the strongest predictor of BMD; because the DASH score emphasizes low-fat dairy product intake and limits sodium intake. Intake of low-fat dairy products has been considered to be associated with improved BMD (34), and intake of lower sodium reduces urinary calcium excretion, which contributes to the bone health (35). However, this relationship between DASH score and BMD has not been significant in children with stimulant use, which may be related to the smaller sample size included (*n* = 222); moreover, we speculated that stimulant use affects the effect of DASH patterns on bone. More studies with larger sample sizes are needed to validate our findings.

Compared with previous studies, our study showed the advantage and highlighted the influence of DPs and PA on BMD and BMC, and osteoporosis in children with stimulant use, which might be of higher practical value to enhance the bone content and decrease the occurrence of osteoporosis. However, our study does have limitations. First, the present research had a relatively small sample size of children with stimulant use, this could be due to the fact that our sample data was derived from the NHANES database. Moreover, the cross-sectional design of the study, cannot demonstrate the causality between DPs and PA on BMC, BMD, and osteoporosis, as a consequence, more trials still are needed to explore this association.

## Conclusion

Improved adherence to MD, DASH, AHEI, or increased PA may increase BMD, and BMC and reduce the risk of osteoporosis; the children with stimulant use should improve adherence to MD and do more PA compared to the children without stimulant use.

## Data availability statement

Publicly available datasets were analyzed in this study. This data can be found here: NHANES database, https://wwwn.cdc.gov/nchs/nhanes/.

## Ethics statement

Ethical approval was not provided for this study on human participants because the data was from a public database. Written informed consent to participate in this study was provided by the participants’ legal guardian/next of kin.

## Author contributions

QL and JZ designed the study, collected, analyzed, and interpreted the data. QL wrote the manuscript. JZ critically reviewed, edited, and approved the manuscript. Both authors read and approved the final manuscript.

## Footnotes

1
https://www.cdc.gov/nchs/nhanes/index.htm
